# Retraction Note: Chinese English Language learners’ vocabulary retention: investigating the effectiveness of neuro/metacognitive and socio-cultural strategies

**DOI:** 10.1186/s40359-025-02811-z

**Published:** 2025-05-06

**Authors:** Wei Hu, Yipeng Luo

**Affiliations:** 1 School of Foreign Language, Hunan University of Science and Engineering, Yongzhou, 425199 Hunan China; 2 School of Information Science and Engineering, Hunan Women& apos; University, Changsha, 410004 Hunan China

**Retraction Note: BMC Psychology (2024) 12:113**

10.1186/s40359-024-01612-0

The editor and the publisher have retracted this article. The article was submitted to be part of a guest-edited issue. An investigation by the publisher found a number of articles, including this one, with a number of concerns, including but not limited to compromised editorial handling and peer review process, inappropriate or irrelevant references or not being in scope of the journal or guest-edited issue. Based on the investigation’s findings the editor therefore no longer has confidence in the results and conclusions of this article.

Wei Hu has stated that the authors disagree with this retraction.

